
JOURNAL INFORMATION
==============================
NLM Title Abbreviation: ScientificWorldJournal
Journal ID: ScientificWorldJournal
NLM Journal ID: 101131163
Journal ID: TSWJ

The Scientific World Journal

ISSN: 2356-6140
EISSN: 1537-744X
Publisher: Wiley

ARTICLE INFORMATION
==============================
PMCID: PMC6084016
PMID: 30147571
DOI: 10.1100/tsw.2001.199
Article ID: 193575
Article version: 1
Subjects: Short Report

Genetic Mapping of Cell Survival Pathways using Enhanced Retroviral Mutagens

Liu Dan
Yang Xiaohong
Songyang Zhou *
Department of Molecular Biology and Biochemistry, Baylor College of Medicine, One Baylor Plaza, Houston, TX 77030, USA
*Zhou Songyang: songyang@bcm.tmc.edu
Publication date: 2001
Electronic publication date: 2001 Jun 5
Volume: 1
First page: 95
Last page: 95
Copyright: Copyright © 2001 Dan Liu et al.
Copyright year: 2001
License: This is an open access article distributed under the Creative Commons Attribution License, which permits unrestricted use, distribution, and reproduction in any medium, provided the original work is properly cited.
License URL: https://creativecommons.org/licenses/by/3.0/

Funding: Ellison Medical Foundation

==============================
Miami *Nature Biotechnology* Short Reports
*TheScientificWorld* (2001) 1 (S3), 95SR
ISSN 1532-2246; DOI 10.1100/tsw.2001.199

### GENETIC MAPPING OF CELL SURVIVAL PATHWAYS USING ENHANCED RETROVIRAL MUTAGENS

Dan Liu, Xiaohong Yang, Zhou Songyang*

Department of Molecular Biology and Biochemistry, Baylor College of Medicine, One Baylor Plaza, Houston, TX 77030
* songyang@bcm.tmc.edu

**INTRODUCTION.** Apoptosis and survival play a crucial role in mammalian development. However the signaling pathways for survival are much less well understood compared to apoptosis pathways. The mammalian cell survival factors known to date have been largely identified either biochemically or based on genetic studies in other organisms. Efficient and effective genetic methods that allow systematic studies of anti-apoptosis genes in mammalian cells remain to be established.

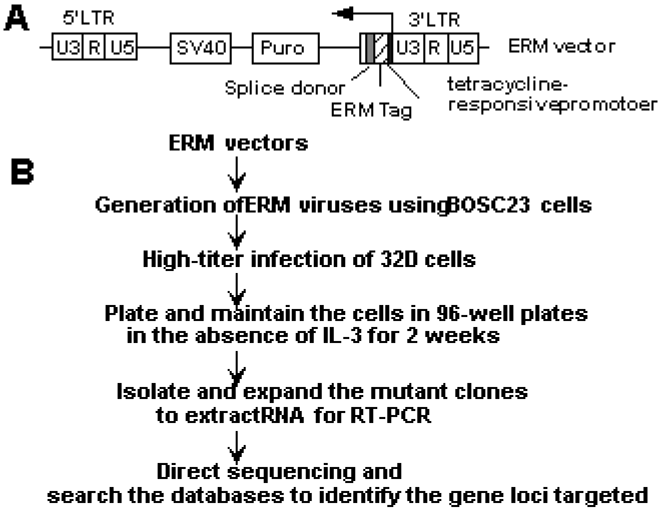

**METHODS.** To identify anti-apoptosis genes throughout the mammalian genome, we utilized a novel retrovirus-based genetic screen method to identify factors that mediate IL-3-dependent survival signaling in hematopoietic cells using enhanced retroviral mutagens (ERM) (1,2). ERM vectors contain several engineered sequences (an ERM tag and a splice donor) controlled by a tetracycline-responsive promoter. Endogenous genes can thus be randomly activated and tagged in a conditional system.

**RESULTS.** A large number of both known and novel cell survival mediators were identified through the screen, including Bcl-$x_L$, PI-3 kinase, and Akt (3,4). One of the survival genes identified is Cytokine Independent Survival Kinase (CISK), a PX-domain containing Ser/Thr kinase homologous to SGK (1). We showed that CISK acts downstream of the PI-3 kinase cascade and may function in parallel to Akt. Similar to Akt, CISK can also phosphorylate *in*

*vivo* Bad and FKHRL1, two known physiological mediators of apoptosis (4). However, CISK exhibits distinct subcellular localization, suggesting that CISK may act in different signaling cascades compared to Akt.

**DISCUSSION.** Our results demonstrate the power of the ERM approach at identifying key genes involved in cell survival and apoptosis signaling without the need of cDNA library construction. The identification of CISK presents a new branch point in PI-3 kinase regulated survival pathways. CISK is the first member of the SGK family proteins to be shown to promote cell survival, pointing to an interesting possibility that other SGK family members may also function in survival pathways.

**ACKNOWLEDGEMENT.** This work was supported by Ellison Medical Foundation (to Z.S.).

**REFERENCES.**

1. 1. Liu, D., Yang, X., and Songyang, Z. (2000) Curr. Biol. 10, 1233-1236
2. 2. Liu, D., Yang, X.H., Yang, D.L., and Songyang, Z. (2000) Oncogene (in press)
3. 3. Vaux, D.L. and Korsmeyer, S.J. (1999) Cell 96, 245-254
4. 4. Datta, S.R., Brunet, A., Greenberg, M.E. (1999) Genes Dev. 13, 2905-2927
